# Comparison of lateral parapatellar vs. infrapatellar approaches for intramedullary nailing for tibial shaft fractures

**DOI:** 10.3389/fsurg.2025.1521860

**Published:** 2025-04-23

**Authors:** Lifeng Yang, Guanghua Nie

**Affiliations:** ^1^ Department of Orthopedics, Luonan County Hospital, Shangluo, Shaan’xi, China; ^2^ Department of Orthopaedics, Honghui Hospital, Xi’an Jiaotong University, Xi’an, Shaan’xi, China

**Keywords:** tibial shaft fractures, lateral parapatellar entry, infrapatellar, intramedullary nailing, surgical approaches

## Abstract

**Background:**

This study aimed to evaluate the clinical and functional outcomes of tibial shaft fractures treated with intramedullary nailing (IMN) using the lateral parapatellar entry (LPE) and infrapatellar (IP) surgical approaches.

**Methods:**

A total of 85 patients with tibial shaft fractures treated with IMN between January 2019 and December 2022 were retrospectively analyzed. A total of 40 and 45 patients underwent IMN using the LPE and IP surgical approaches, respectively. The operation time, intraoperative fluoroscopy times, blood loss, closed reduction rate, fracture healing time and complications were reviewed in this study. The American Orthopaedic Foot and Ankle Society (AOFAS) scale and Lysholm Knee Scoring Scale were used as functional measurements.

**Results:**

The study included 85 patients (40 in the LPE group and 45 in the IP group), with a minimum follow-up of 12 months. No significant differences were found in fracture healing time, closed reduction rate, infection, deformity healing rate, Lysholm scores, and AOFAS scores between the groups. The LPE group displayed an significantly shorter operation duration, less blood loss, fewer fluoroscopy times, and a lower average VAS score compared to the IP group (*P* < 0.05).

**Conclusions:**

The LPE approach for IMN in tibial shaft fractures may offer advantages in terms of fewer fluoroscopy times, and lower complication rates, suggesting it could be a preferable surgical approach.

## Introduction

Tibial shaft fractures are among the most prevalent long bone fractures, often resulting from high-energy trauma such as motor vehicle accidents or falls from significant heights (1). These fractures present a therapeutic challenge due to the wide variety of fracture patterns and the potential for significant soft tissue injury (1). Intramedullary nailing (IMN) has become the gold standard for the surgical treatment of these fractures, offering advantages such as early weight-bearing and a lower risk of malunion compared to other methods like external fixation and plating (2). The intramedullary nail can be inserted through different surgical approaches, primarily the infrapatellar (IP), suprapatellar (SP) and the lateral parapatellar entry (LPE) approaches. The choice of approach can significantly impact the clinical outcomes, including the healing time, functional recovery, and complication rates (2, 3). The IP approach, traditionally more common, involves inserting the nail through an incision below the patella, which can be technically demanding and may cause anterior knee pain with a variable incidence ranging from 28.6% to 65% (4). The SP approach involves inserting the nail through an incision above the patella, purportedly offering better alignment and less anterior knee pain, but it needs special instruments (5). Kubiak et al. reported a novel technique using the semi-extended position while performing a lateral parapatellar as extra-articular approach during tibial nail placement (6). Several studies reported that patients with tibia fractures who were treated with IMN using the LEP and IP approaches have similar functional outcomes for tibial shaft fractures (7, 8). However, despite these findings, a consensus on the LEP approach remains elusive due to variability in study designs and patient populations.

This study aimed to compare the clinical and functional outcomes of tibia shaft fractures treated with IMN using the LPE and IP surgical approaches. By providing a comprehensive comparison, this study seeks to inform clinical decision-making and optimize treatment strategies for tibial shaft fractures.

## Methods

This retrospective study was approved by the Ethics Committee of the Luonan county hospital. This retrospective study data from patients who underwent tibial shaft fracture treated with IMN between January 2019 and December 2022. The inclusion criteria were as follows: age >18 years, closed fractures, Fresh tibial shaft fractures (AO/OTA type 42 A-C) and longer than 12 months follow-up. The exclusion criteria were as follows: open fracture, pathological fractures, knee stiffness and patients with diseases (chronic kidney disease, albumin <35 g/L, severe anaemia). Overall, 40 patients treated with IMN through the LPE approach (group LPE) and 45 patients treated with IMN through the traditional IP approach met the inclusion and exclusion criteria of this study. There were no statistical differences in demographic data between the two groups (Table 1). The senior orthopedic surgeons conducted the surgeries using the LEP approach technique described by Stella et al. (9) or the IP approach technique (IP group) described by Lu et al. (10). The patellar tendon was longitudinally incised in all patients for IP group, and transtendinous access was established. The infrapatellar fat pad was meticulously cleaned to reveal the tibial plateau slope, and the appropriate nail entry point was identified in relation to the medullary cavity. The knee was flexed to 90°, and an incision was performed. After manual reduction, the assistant sustained the reduction. The tibia IMN guide was introduced, and the medulla was expanded. Once the medulla was fully expanded, an IMN was positioned into the articular surface of the distal tibia by approximately 1 cm. The fracture alignment, main nail thickness, and depth were assessed from the C-arm viewpoint. Following satisfactory reduction, the fracture was stabilized with proximal and distal locking screws. If the closed reduction proved to be challenging, a minor incision was created at the site of the fracture to assist the reduction. A pre-shaped foam ramp or towel/blanket incline is positioned underneath the affected limb, ensuring that the hip and knee are bent at approximately 30° for LEP group. A 3 cm lateral incision is made to the lateral tibial spine at the anterior articular margin. The remaining operations is the same as for the IP approach technique.

**Table 1:** Demographic data of the two groups.

Characteristics	Total	LPE	IP	*P*
Age (years)	40.7 ± 8.1	41.3 ± 6.5	40.1 ± 5.7	0.367
Gender (M/F)	55/30	25/15	30/15	0.821
AO classification (42 A/42 B/42 C)	（27/35/23）	（14/17/9）	（13/18/14）	0.493
Time to surgery (day)	3.4 ± 1.1	3.3 ± 1.2	3.5 ± 1.1	0.425
Follow-up (month)	20.2 ± 2.1	20.1 ± 2.9	21.3 ± 3.1	0.069

The operation time, number of surgeons, intraoperative fluoroscopy times, blood loss, closed reduction rate, fracture healing time and complications were extracted from the medical record in this study. The knee functional measurements were evaluated using the Lysholm Knee Scoring Scale (11), and the ankle functional measurements were evaluated using The American Orthopaedic Foot and Ankle Society (AOFAS) scale (12) at the final follow-up. The visual analog scale (VAS) was used to evaluate patients' pain at the final follow-up. Fracture deformity was defined as fracture shortening or parallel displacement >5 mm, anterior-posterior or medial-lateral angle >5°, and a rotation angle >10° (13).

Statistical analysis was performed using GraphPad Prism 8.0. The Shapiro–Wilk test was first used to determine whether the data were normally distributed. Quantitative data that conform to normal distribution were expressed as mean (standard deviation), and t-tests were used for inter group comparison. Count data is expressed as a percentage, and intergroup comparisons are conducted using the *χ*^2^ test. The categorical data was performed using *χ*^2^ test. *P* < 0.05 is considered statistically significant for the difference.

## Results

### Characteristics of patient demographics

After applying the inclusion and exclusion criteria, a total of 85 patients (40 LPE vs. 45 IP) were included in this study. The average age was 40.7 ± 8.1 years. The average follow-up time was 20.2 ± 2.1 months. Patients' characteristics data, including age, sex, fracture type, time to surgery, AO/*OTA classifcation* and follow-up time were comparable (*P* > 0.05) (Table 1).

### Surgical comparison between the two groups

The average operation duration for the LPE group and IP group was 80.21 ± 8.84 min and 89.34 ± 11.25 min, respectively, with a statistically significant difference observed (*p* < 0.001). The frequency of intraoperative fluoroscopy in the LPE group and IP group was 17.82 ± 2.80 times and 22.46 ± 2.97 times, respectively, indicating a significant difference (*p* < 0.001). The average blood loss during surgery in the LPE group and IP group was 62.3 ± 9.9 ml and 69.5 ± 16.3 ml, respectively, indicating a significant difference (*p* = 0.017). The average number of surgeons was 2 (1–3) in LPE group and 3 (1–4) in IP group (*P* < 0.001). Closed reduction rate was employed in 30 patients (75.0%) in the LPE group and 31 patients (68.9%) in the IP group, revealing no significant difference between the two groups (*p* = 0.868) (Table 2).

**Table 2:** Surgical and prognostic comparison of the two groups.

Characteristics	LPE	IP	*P*
Operation time (min)	80.21 ± 8.84	89.34 ± 11.25	<0.001
Fluoroscopy time (s)	17.82 ± 2.80	22.46 ± 2.97	<0.001
Blood loss (ml)	62.3 ± 9.9	69.5 ± 16.3	0.017
Closed reduction rate (%)	90% (36/40)	88.9% (40/45)	0.868
Number of surgeons (N)	2 (1–3)	3 (1–4)	<0.001
Fracture healing (week)	16.9 ± 4.2	16.2 ± 5.9	0.535
AOFAS score	91.3 ± 9.2	92.1 ± 7.2	0.654
Lysholm knee score	88.9 ± 5.9	86.1 ± 7.2	0.055

### Prognostic comparison

**Figure 1:**
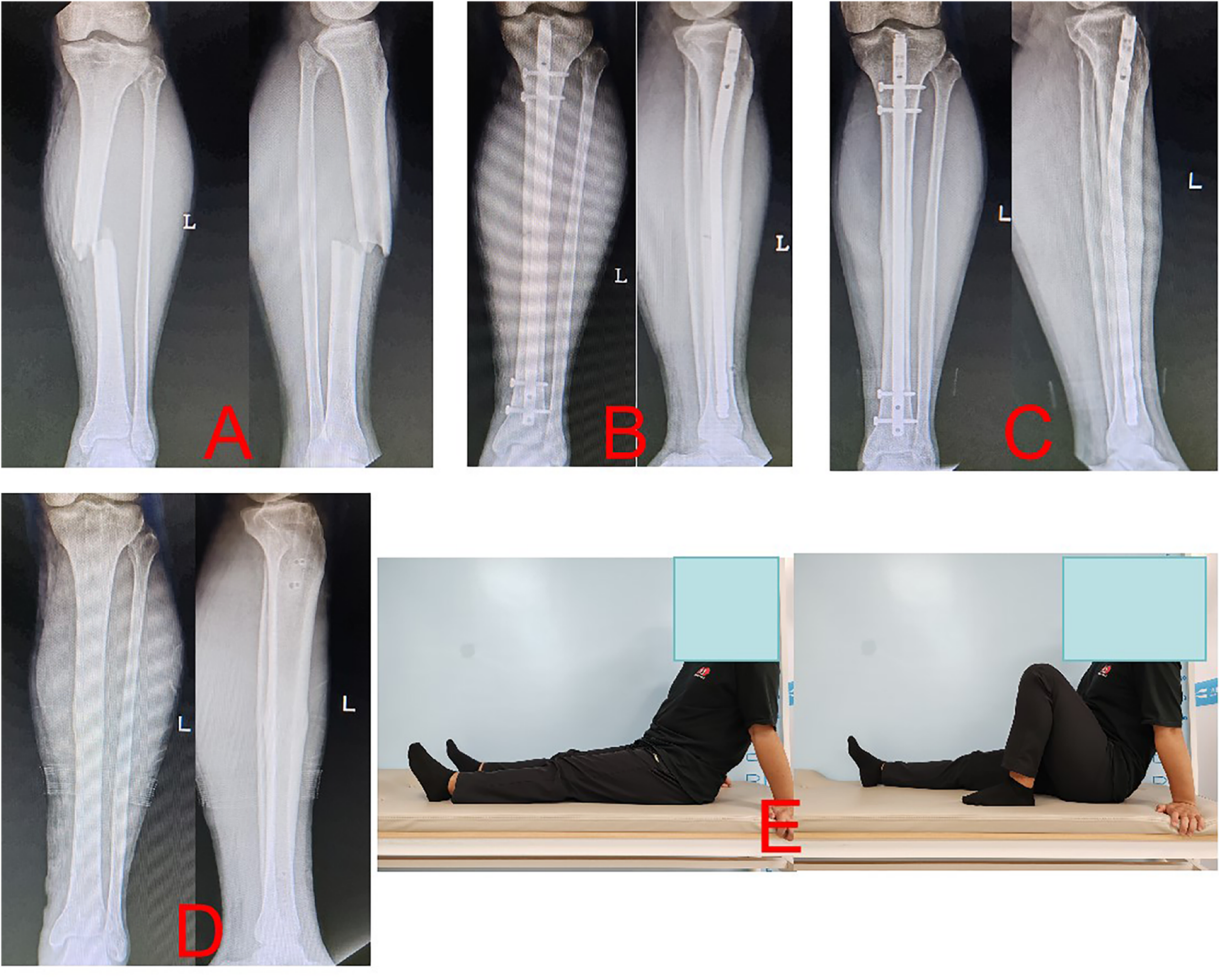
Radiographs of a case (a 33-year old man with the left tibia shaft fracture (AO- 42A3) of union after closed reduction using the LPE approach were presented. **(A)** Preoperative anteroposterior (AP) and lateral views. **(B)** AP and lateral views postoperatively. **(C)** AP and lateral views 13 months postoperatively. **(D)** AP and lateral views after IMN was removed. **(E)** Functional recovery at 13 months postoperatively.

**Figure 2:**
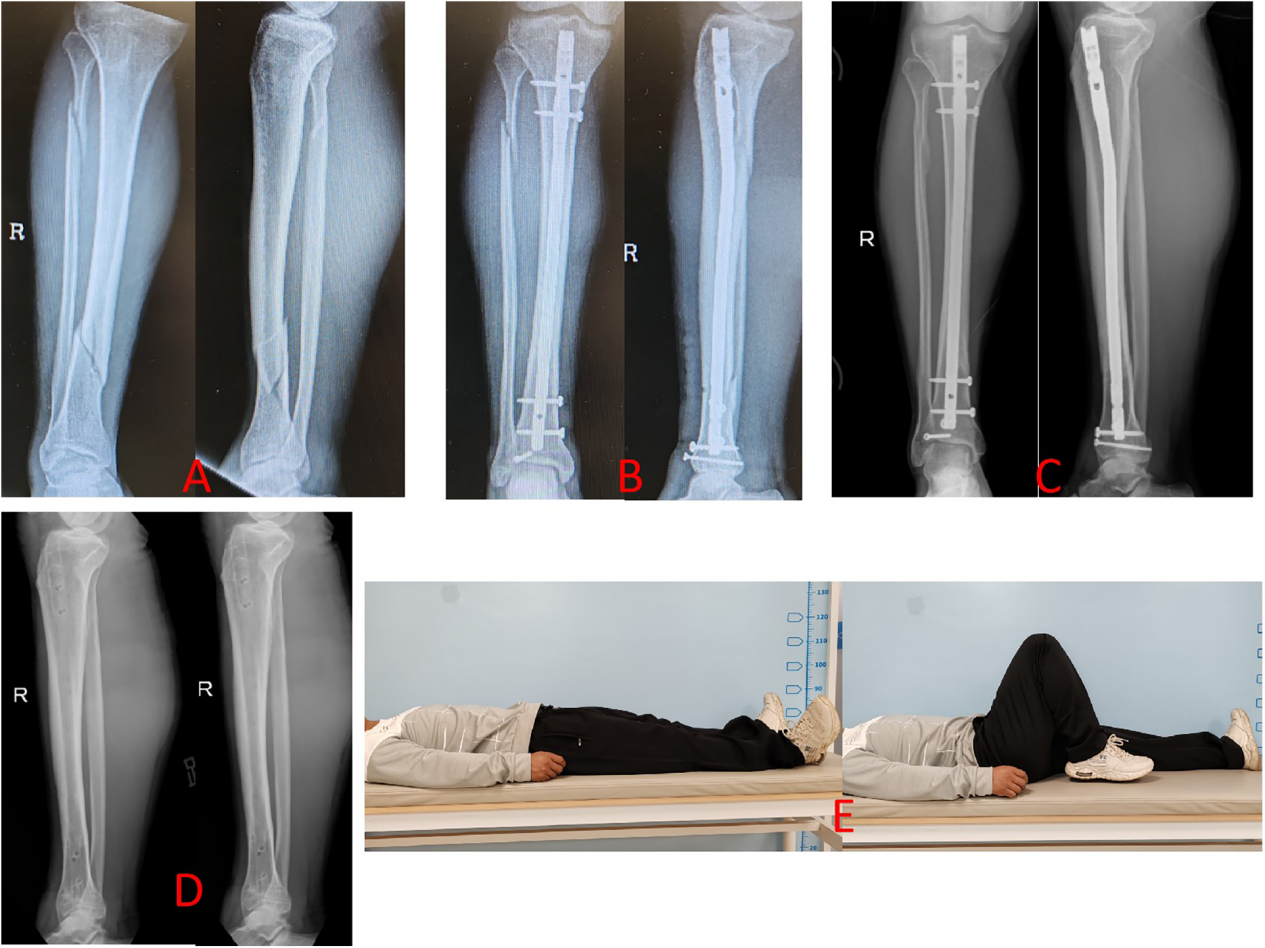
Radiographs of a case (a 29-year old man with the right tibia shaft fracture (AO- 42B3) of union after closed reduction using the IP approach were presented. **(A)** Preoperative anteroposterior (AP) and lateral views. **(B)** AP and lateral views postoperatively. **(C)** AP and lateral views 12 months postoperatively. **(D)** AP and lateral views after IMN was removed. **(E)** Functional recovery at 12 months postoperatively.

The mean fracture healing duration was 16.9 ± 4.2 weeks in the LPE group (Figure 1) and 16.2 ± 5.9 weeks in the IP group (2) (Figure 2), showing no significant difference (*p* < 0.05). The Lysholm score and AOFAS score were 88.9 ± 5.9 and 91.3 ± 9.2 in the LPE group, and 86.1 ± 7.2 and 92.1 ± 7.2 in the IP group, respectively, with no significant difference (*p* = 0.055 and *p* = 0.654) (Table 2).

### Comparison of complications

The infection rate in the LPE group was 5.0% (2/40) and 8.8% (4/45) in the IP group, there was no notable difference between the two groups (*P* = 0.485). No deep infections were observed in either group. The deformity healing rate was 7.1% (3/40) in the LPE group, which was inferior to that in the IP group [13.3%, (6/45)]; however, the difference was not statistically significant (*P* = 0.383). The mean VAS score was significantly reduced in the LPE group (0.4 ± 0.9 vs. 1.2 ± 1.3; *P* = 0.001) in comparison to the IP group (Table 3).

**Table 3:** Complications of the two groups.

Characteristics	LPE	IP	*P*
Infection (%)	5.0 (2/40)	8.8 (4/45)	0.485
Deformity healing rate (%)	7.1 (3/40)	13.3 (6/45)	0.383
VAS	0.4 ± 0.9	1.2 ± 1.3	0.001

## Discussion

Tibia shaft fractures are prevalent orthopedic injuries, often associated with high-energy trauma such as vehicular accidents. These fractures can lead to severe pain, functional impairment, and a marked decrease in quality of life for patients, while also imposing significant economic burdens on healthcare systems due to high treatment costs and potential complications during recovery (2). Surgical interventions, particularly intramedullary nailing, have become standard practice for managing these fractures. However, the complexity of surgical techniques and varied recovery outcomes highlight the need for further research to optimize treatment modalities and enhance patient outcomes (14).

In this study, we investigated the clinical outcomes of tibial shaft fractures treated with IMN using two different surgical approaches: the LPE and the IP. Our findings revealed that both approaches are effective in treating tibial shaft fractures, with specific differences in surgical and postoperative outcomes. While the LPE method demonstrated a shorter operative time, fewer fluoroscopic exposures and less blood loss compared to the IP group, which suggests a potentially more efficient surgical process. The LPE technique was one of the semi-extension postures. Previous studies indicated that the semi-extension postures can diminish radiation exposure duration and operative time (15, 16). Furthermore, the functional outcomes assessed through the Lysholm knee scoring and AOFAS scoring systems indicated comparable results between the two groups. This novel insight suggests that when selecting a surgical method for tibial shaft fractures, the LPE could be prioritized due to its operational efficiency, without sacrificing patient outcomes.

This study revealed that the VAS pain score was markedly reduced in the LPE group compared to the IP group. The incidence of anterior knee pain was higher in the IP group. Several studies indicated that the prevalence of anterior knee pain following IP approach varies from 10% to 80% with a mean of 47.4% (17, 18). Marco Stella et al. demonstrated that the occurrence of anterior knee pain was minimal and virtually insignificant in a prospective investigation of the lateral parapatellar extra-articular technique (9). In this study, the VAS pain score in the LPE group was 0.4 ± 0.9, which was significantly lower than 1.2 ± 1.3 in the IP group (*P* = 0.001). This finding supports the hypothesis that while both surgical approaches are viable, the LPE approach may offer certain advantages in terms of anterior knee pain, thereby influencing the choice of surgical technique based on patient-specific factors and surgeon expertise.

Malalignment is regarded as one of the primary complications of IMN treatment for tibial shaft fractures. Malalignment of the tibia not only alters the tibial alignment and the typical stress distribution of the ankle joint, but research has also demonstrated that even a 5°malunion can result in ankle pain and subtalar joint stiffness (19). Consequently, enhancing the quality of fracture reduction is the most efficacious approach to minimize malunion and enhance long-term functionality. Lu et al. demonstrated that a notable disparity existed in the rate of malalignment following IMN treatment of tibial fractures via the semi-extended SP approach compared to the IP approach, with the semi-extended SP approach group exhibiting superior outcomes relative to the IP approach group (10). The LPE method and the SP method are both semi-extension techniques and are expected to yield comparable outcomes for malalignment. The semi-extended LPE method does not necessitate limb position modification, which facilitates the preservation of fracture alignment and diminishes the likelihood of re-displacement following reduction (20). In this study, the deformity healing rate was 7.1% (3/40) in the LPE group, which was inferior to that in the IP group [13.3%, (6/45)], however, the difference was not statistically significant (*P* = 0.383).

Traditionally, the IP technique is the most commonly employed approach. However, for proximal third fractures, flexion beyond 30° tends to elongate the fracture line, resulting in an apex anterior misalignment of the fracture. The additional IP technique need Poller screws and/or K-wires to reduce canal diameter for proximal and distal tibial fractures (21). Presently, the application of SP technique nails for proximal third fractures is highly endorsed, as the meticulous calibration of the nail entry point is more manageable, and the semi-extended position of the knee facilitates the reduction of the fracture without additional measures (22). The SP technique is linked to a markedly improved functional outcome, reduced knee pain, and a diminished incidence of fracture deformity compared to the IP IMN technique in the management of distal tibia fractures (10). The LPE method is semi-extension technique. So, the LPE technique method is more suitable for treatment proximal and distal tibial fractures.

However, it is crucial to acknowledge the limitations of our study, primarily stemming from its retrospective design and relatively small sample size. This may limit the generalizability of our findings across broader populations. Additionally, the lack of a randomized controlled trial design might introduce selection biases that could affect the validity of the results. Future investigations should aim for larger, multicenter randomized controlled trials to solidify these findings and explore long-term outcomes associated with both surgical approaches more comprehensively. Such studies would be instrumental in further refining surgical techniques and ensuring best practices in the treatment of tibial shaft fractures.

In conclusion, this study provides valuable insights into the comparative effectiveness of the LPE approach and traditional IP approach in the surgical management of tibial shaft fractures. The findings reveal that while LPE demonstrates advantages in terms of surgical duration and postoperative pain management, both approaches yield comparable results regarding fracture healing times and functional recovery. These results underscore the need for clinicians to consider individual patient characteristics when selecting surgical techniques. Although the study contributes to the existing literature, further large-scale randomized controlled trials are necessary to confirm these findings and refine clinical practice guidelines in the treatment of tibial shaft fractures.

## Data Availability

The original contributions presented in the study are included in the article/Supplementary Material, further inquiries can be directed to the corresponding author.
